# Efficacy of a unique omega-3 formulation on the correction of nutritional deficiency and its effects on cardiovascular disease risk factors in a randomized controlled VASCAZEN^®^ REVEAL Trial

**DOI:** 10.1007/s11010-014-2132-1

**Published:** 2014-09-04

**Authors:** Nisar A. Shaikh, Jason Yantha, Sabah Shaikh, William Rowe, Maggie Laidlaw, Carla Cockerline, Abbas Ali, Bruce Holub, George Jackowski

**Affiliations:** 1 University of Toronto, 6190 Tremaine court, Mississauga, ON L5V 1B5 Canada; 2 Pivotal Therapeutics Inc., 81 Zenway Blvd, Woodbridge, ON L4H 0S5 Canada; 3 Nutrasource Diagnostics Inc., 120 Research Lane, Guelph, ON N1G 0B4 Canada; 4 Florida Cardiology Clinic, 255 Citrus Towers, Clermont, FL 34711 USA

**Keywords:** Nutrition, Omega-3 (n-3) polyunsaturated fatty acids, Omega-3 deficiency, Lipids and lipoproteins, cardiovascular disease, Biomarkers, Randomized control trials

## Abstract

Low blood levels of long chain omega-3 polyunsaturated fatty acids (LC n-3 PUFA) have been reported to be associated with increased risk for cardiovascular disease (CVD) deaths. Systematic studies measuring LC n-3 PUFA blood levels (pre and post-treatment) in defined subjects, and monitoring the correction of nutritional deficiency with a pure LC n-3 PUFA formulation in sufficient doses, while monitoring CVD risk factors are lacking. We tested the efficacy of a novel LC n-3 PUFA Medical Food formulation (VASCAZEN^®^, > 90 % pure with a 6:1 eicosapentaenoic acid-(EPA):docosahexaenoic acid-(DHA) ratio; 6:1-OM3), to correct such deficiency and determine the concomitant effects on lipid profiles. Of 655 subjects screened, 89 % were LC n-3 PUFA deficient (Omega-Score, (OS) = blood EPA + DHA + Docosapentaenoic acid < 6.1 %). From these, a study was conducted on 110 ambulatory cardiovascular subjects. Placebo: corn oil. Primary endpoint: change in OS. Secondary endpoint: changes in blood lipid profiles. At 8 weeks of treatment with 6:1-OM3 (4 g/day), placebo-adjusted median OS levels (*n* = 56) significantly improved (132 %, *P* < 0.0001) with a decrease in AA (arachidonic acid): EPA ratio (82 %, *P* < 0.0001). In hypertriglyceridemic subjects (TG 2.26–5.65 mmol/L), HDL-C improved (9 %, *P* = 0.0069), TG-reduced (48 %, *P* < 0.0001), and VLDL-C reduced (30 %, *P* = 0.0023), without significantly affecting LDL-C levels. This study confirms that LC n-3 PUFA deficiency is prevalent in the US population, and its correction with 6:1-OM3 in CVD subjects improves lipid profiles. The purity, EPA:DHA ratio and dose are determinant factors for optimal efficacy of a formulation in reducing CVD risk factors.

## Introduction

The cardiovascular health benefits of a diet rich in fish has been known for decades and such benefits have been attributed to the presence of LC n-3 PUFA found in fish [1–4]. The typical Western Diet is relatively deficient in LC n-3 PUFA but enriched in saturated fat and n-6 PUFA [5], leading to chronic LC n-3 PUFA deficiency in the US population. About 70 % of the US population, assessed on the basis of dietary intake, is deficient in LC n-3 PUFA blood levels [6]. Such deficiency can be determined through the measurement of EPA + DHA + DPA (docosapentaenoic acid) levels in blood (Omega-Score™, OS) or of EPA + DHA levels in red blood cells (Omega-3 Index, OI) [7, 8]. Although cut-off levels for OS or OI have yet to be defined, LC n-3 PUFA dietary deficiency or insufficiency has been identified as a risk factor attributable to 84,000 all cause deaths per year in the US [9]. In a prospective trial, through analyses of blood samples from the Physicians’ Health Study, the lowest quartile of LC n-3 PUFA has been correlated to about 81 % increased risk of sudden cardiac death among men without prior evidence of CVD [10]. Based upon “risk quartiles” of OS levels, the authors concluded that an OS >6.1 % is strongly associated with a reduced risk for sudden cardiac death. Similar findings were reported earlier where a strong inverse relationship between OI and primary cardiac arrest was described [11]. More recently, quintiles of individual EPA, DHA,and DPA levels have been shown to be associated with lower cause-specific CVD mortality and their combined levels were associated with a 35 % lower risk [12]. Furthermore, the clinical utility of the OI has been suggested not only as a biomarker of intake but also as an important risk factor for CVD and target for therapy [8, 13].

Despite the known beneficial effects of LC n-3 PUFA, a number of meta-analyses of randomized controlled trials (RCT) have generated controversy for their use as a preventive treatment option for CVD subjects [14–17]. Some of the studies included had study design deficiencies that may have contributed to outcomes. The treatments employed were often either of low purity with varying ratios of EPA and DHA, and/or doses were suboptimal. Importantly, many studies that employed high purity supplements did not measure OS or OI levels either at pre- or at post-treatment [18–20]. In other studies, either baseline EPA levels (2.9 % of the total fatty acids) were high [19], or fish consumption increased significantly during the study [21] that may have undermined the beneficial effects of the treatment. Others have often included patients on concomitant medications (though unavoidable), which can possibly affect the trial outcomes [22–24]. For example, amiodarone, an antiarrhythmic agent, has been shown to be a potent phospholipase inhibitor [25], an enzyme needed for the fatty acid turnover (acylation/de-acylation cycle) in cell membranes. In addition, the choice of placebo employed could also affect placebo-corrected results [26]. The importance of these limitations and emphasis on patient selection, individual patient data meta-analysis, insufficient dose,and purity of supplement have been emphasized in recent reviews [17, 27, 28]. With the exception of von Schacky and Harris [7, 8, 29, 30] who proposed the OI as an important marker of CVD risk, investigations of the prevalence of LC n-3 PUFA nutritional deficiency and its correction, with health-promoting effects that correlate with increased EPA and DHA intake are lacking.

The primary objective of the present RCT was to investigate the efficacy of a highly pure and a novel 6:1-OM3 formulation to correct LC n-3 PUFA deficiency and improve lipid profiles in ambulatory CVD subjects. To accommodate suggestions proposed in previous reviews [17, 27, 28], the present study (a) selected the subjects with LC n-3 PUFA insufficiency (OS < 6.1 %) with normal and high TG (1.02–5.65 mmol/L, representing 90–500 mg/dL) levels, (b) employed high doses of EPA + DHA (3.2 g/d) with >90 % purity in a novel 6:1 EPA:DHA formulation, which has been shown to produce maximum and sustained vasodilatory effects in isolated perfused porcine coronary artery rings than other known EPA alone or EPA:DHA (1.2:1) preparations [31] and finally (c) measured both pre- and post-treatment OS and OI levels to correlate changes with the trial objectives. Preliminary work of this study has been presented [32].

## Experimental design and methods

This study entitled “A Placebo-Cont**R**oll**E**d Study of **V**ASCAZEN^®^ in Subjects with D**E**ficient Blood LC n-3 Fatty **A**cid **L**evels”, the VASCAZEN^®^-REVEAL Trial, was an external contract research organization (CRO)-facilitated study managed by Nutrasource Diagnostics Inc., Guelph, Ontario, Canada, and conducted at two centers in the United States. The CRO was responsible for all trial operations, including research ethics board communications and protocol approvals, staff training, site visits, data management, and they followed current Good Clinical Practice, the Declaration of Helsinki guidelines, and research ethics board guidelines and requirements. The study protocol was approved by the Canadian SHIELD Ethics Review Board. Pivotal Therapeutics Inc. (Woodbridge, Ontario, Canada) sponsored the study.

This 8-week study was a multicenter, randomized, double-blinded, placebo-controlled study that investigated LC n-3 PUFA nutritional deficiency and its correction with 4 g/day of corn oil or 6:1-OM3 formulations (Supplied by Pivotal Therapeutics Inc). Ambulatory cardiovascular subjects were questioned and screened for their eligibility into the trial. Male and female study subjects ≥18 years of age, with one or more risk factor for CVD, were deemed eligible for study enrollment if their fasting whole blood OS levels were <6.1 % by weight of total blood fatty acid levels, and their serum TG was between 1.02 and 5.65 mmol/L. Subjects were excluded from the study if they refused to provide informed consent, had a known allergy to fish, were premenopausal women, were currently taking hormone replacement therapy (HR), lipid-altering medication or LC n- PUFA supplements, had a history of alcohol abuse, were medically ill, had a history of ventricular arrhythmia, bleeding or clotting disorder, liver or kidney disease, autoimmune disorder or suppressed immune systems, myopathy or rhabdomyolysis, seizure disorder, or had an implantable cardioverter defibrillator. Subjects on a stable statin medication for a minimum of three months were eligible to enroll.

All eligible subjects were randomized using a list generated by the CRO (random assignment of “Treatment A”, or “Treatment B”), to receive four 1 g capsules per day of either corn oil (placebo) or 6:1-OM3 (providing a minimum of 2.72 g/day of EPA and 440 mg/day of DHA). Corn oil was almost completely devoid of LC n-3 PUFA and mostly consisted of n-6 & n-9 fatty acids (linoleic 59 %; oleic 25 %; palmitic 11 %; stearic 2 %), while 6:1-OM3 contained over 94 % LC n-3 PUFA and less than 5 % n-6 fatty acids. Both treatment capsules were indistinguishable from one another, and packaged by a third party in white identical boxes labeled “Treatment A” or “Treatment B.” Subjects were stratified by their baseline TG levels into two Cohorts. Cohort 1 included subjects with normal to marginally high TG levels (1.02–2.25 mmol/L representing 90–199 mg/dL) and Cohort 2 included subjects with hypertriglyceridemia (2.26–5.65 mmol/L representing TG 200–500 mg/dL). Randomization was on a 1:1 basis to ensure approximately equal numbers of subjects in each of the two treatment arms. All investigators, the CRO, and the study sponsor, remained blinded to the treatment regimen during the study’s progress.

The primary end point of the study was to determine the efficacy of the 6:1-OM3 treatment to correct LC n-3 PUFA deficiency, by measuring the placebo-adjusted change in blood OS and OI levels post-treatment. Secondary outcome measures included changes in serum levels of TG, VLDL-C, IDL-C, LDL-C, HDL-C, apo-A, apo-B, total cholesterol (TC), high sensitivity C-reactive protein (hs-CRP), arachidonic acid (AA):EPA ratio, n-6:n-3 ratio, and fasting blood glucose from the study baseline to the study end point after 8 weeks of treatment.

### Treatment schedule and subject evaluation

Enrolled subjects provided the baseline 12-hour fasting blood specimens for analyses and completed the health assessment forms. In addition, subject weight, body mass index (BMI), waist and hip circumference and blood pressure were measured and recorded in case report forms. Subsequent to the collection of baseline blood specimens, enrolled subjects were stratified as Cohort 1 or Cohort 2 based upon their fasting TG levels obtained at the screening evaluation. Subjects in each Cohort were provided with 4 capsule/day doses of either Treatment A or B for 8 weeks. All subjects provided 10–12-hour fasting blood specimens by venipuncture at week 0 (baseline) and after 4 and 8 weeks of treatment. The samples were sent for analyses of the primary and secondary measures, as described below. In addition, health assessments (weight, BMI, blood pressure), capsule compliance evaluation, and adverse events reporting were measured, and reported at each subsequent clinic visit. An emergency number was provided to each study participant for reporting any serious adverse reaction during the entire study period.

### Sample analysis

Analyses of total blood fatty acids levels, including OS, OI, AA:EPA ratio, n-6:n-3 ratio and fasting blood glucose were performed at a central laboratory, (University Health Network, Specialty Laboratory, Toronto, Ontario, Canada), accredited by the College of American Pathologists’ Laboratory Accreditation Program. The fatty acid compositions of whole blood were determined on 200 μl of sample after lipid extraction by a modification of the method of Bligh and Dyer [33]. The total lipid fraction was then methylated with 12 % (w/w) boron trifluoride in methanol by incubation at 90 °C for 25 min to produce fatty acid methyl-esters (FAME). After cooling, the FAME were extracted with hexanes, washed with water, dried under nitrogen and dissolved in hexanes. The fatty acid composition was then determined by GLC performed on a 100 m Varian Select™ FAME CP7420 capillary column (0.25 mm i.d.), using an Agilent Technologies 6890 N series gas chromatograph equipped with a split/splitless mode injector, and a flame ionization detector. The injector and detector were maintained at 280 and 300 °C,respectively, and samples were analyzed by multilevel temperature programing in the range of 90–265 °C with ultra high purity grade helium as the carrier gas. The percent composition of fatty acids was calculated from the individual peak areas using appropriate standards. The procedure was routinely validated by proficiency testing using GC/MS. The OS levels were calculated by adding the individual EPA, DPA, and DHA levels. The OI was determined as previously described [13].

Analyses of all other serum analytes, including TG, VLDL-C, IDL-C, LDL-C, HDL-C, apo-A, and apo-B, hs-CRP were performed at Atherotech Diagnostics Lab (Birmingham, Alabama, USA), as described earlier [34].

### Power and sample size

Earlier randomized placebo-controlled studies have indicated that the administration of EPA or DHA therapy over a 6 to 7 week time period can result in an increase in blood LC n-3 PUFA levels of at least 47 % and a decrease in blood TG levels of 18–21 % [35, 36]. Assuming a mean baseline blood OS level of at least 3 % and a change in standard deviation of 1.5 % within a given study stratum [37], a sample size of 40 subjects per study stratum (i.e. 20 subjects assigned to 6:1-OM3 therapy, and 20 subjects assigned to placebo) would result in a minimum power of 90 % to detect an increase in blood OS levels of at least 60 % in subjects within the 6:1-OM3 study arm relative to subjects within the placebo arm, at a significance level of alpha = 0.05 [37]. Assuming a mean baseline blood TG levels of at least 1.58 mmol/L and a change standard deviation of 0.40 mmol/L within a given study stratum, the proposed sample size of 40 subjects per study stratum (i.e. 20 subjects assigned to 6:1-OM3 therapy, and 20 subjects assigned to placebo) would result in a minimum power of 95 % to detect a relative decrease in blood TG levels of at least 30 % in subjects within the 6:1-OM3 study arm relative to subjects within the placebo arm, at a significance level of alpha = 0.05.

### Data analyses

At the conclusion of the study, the Clinical Trial Review Committee assessed the CRF for compliance with the protocol and subjects who ingested a minimum of 80 % of the assigned capsules were locked in. The tabulated data were then un-blinded and analyses performed on the “per protocol” subset of study. The median change and interquartile range in blood OS and OI levels over the 8-week period, as well as all secondary outcome measures was computed for each study subject and organized by subpopulations based on treatment group, and cohorts. Percent changes from baseline to week 8 were calculated for each treatment group (placebo and 6:1-OM3) and placebo-adjusted median percent changes were then calculated. A two-tailed *t* test (unpaired) was performed using GraphPad Prism version 6.0 for Macintosh (GraphPad Software) to illustrate placebo-adjusted changes and significance was defined as a *P* value ≤0.05.

### Safety evaluation

The qualified principal investigators at each site evaluated patient health while remained blinded to the study treatment regiment. All adverse events, defined as “treatment-emergent” (TEAE), which occurred during the study period, were evaluated and classified as related or unrelated to treatment by the principal investigator. Each TEAE was recorded in an individual subject’s case report form at each clinic visit, through patient health evaluations, including a questionnaire, physical examination, clinical laboratory test evaluations, weight, and BMI. Reports were compiled, and categorized based upon grade, severity, and relationship to study treatment. The Clinical Trial Review Committee reviewed the subjects’ case report forms, and agreed with the principal investigator’s assignments.

## Results

### Study description and demographic characteristics of the enrolled subjects

**Fig. 1:**
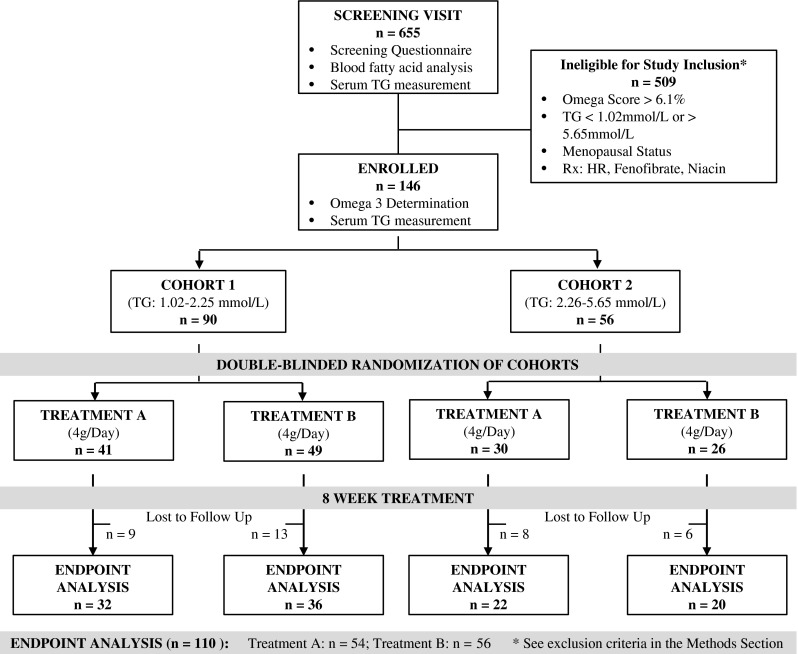
Study design and patient disposition

The study protocol outline is shown in Fig. 1. Of the 655 screened, 509 subjects were ineligible for study enrollment as per inclusion/exclusion criteria in the methods section. The remaining 146 subjects were stratified into two Cohorts and were randomized, blinded and treated with either corn oil (A) or 6:1-OM3 (B). Thirty-six subjects were either lost to follow-up or the compliance with the treatment was less than 80 %. The OS data from the 655 screened subjects showed LC n-3 PUFA nutritional deficiency was prevalent in general US population, with 89 % of subjects falling below 6.1 % OS (median OS 4.48 %; range 1.84–5.89 %).

The baseline characteristics of the enrolled and randomized study population (Table 1) were comparable within Cohorts, and across treatment groups with approximately equal numbers of males and females enrolled. The median baseline LC n-3 PUFA level (measured as OS or OI) or mean serum TG levels were comparable among two Cohorts and treatment groups. The mean BMI was >30 (obese) for all groups and Cohorts. Statin use was also similar, except for placebo group in Cohort 1, which had twice as many subjects on this drug as in other groups. Additional CVD risk indicators such as diabetes, hypertension, and low HDL-C are approximately similarly matched among Cohorts and treatment groups (Table 1).

**Table 1:** Baseline characteristics of the study participants

	Cohort 1		Cohort 2	
	Placebo	6:1-OM3	Placebo	6:1-OM3
	(*n* = 32)	(*n* = 36)	(*n* = 22)	(*n* = 20)
Age (years)	56.0 ± 16.6	53.4 ± 13.9	50.5 ± 11.7	53.5 ± 12.1
Male/Female (numbers)	15/17	21/15	11/11	12/8
Median Omega-Score (interquartile range)	3.38 (0.97)	3.64 (0.94)	3.36 (1.10)	3.15 (0.88)
**Baseline parameters** ^a^				
BMI (kg/m^2^)	31.8 ± 7.0	30.8 ± 7.1	32.5 ± 4.3	33.3 ± 6.7
MAP (mmHg)	93.1 ± 8.5	91.4 ± 9.1	94.4 ± 6.9	94.6 ± 8.5
**Risk factors** ^b^				
Diabetes	6 (18.8 %)	3 (8.3 %)	2 (9.1 %)	3 (15.0 %)
Hypertension	11 (34.4 %)	10 (27.8 %)	6 (27.3 %)	8 (40.0 %)
Patients on a statin	8 (25.0 %)	4 (11.1 %)	4 (18.2 %)	4 (20.0 %)
HDL < 0.78–1.04 mmol/L (Male–Female)	19 (59.4 %)	26 (72.2 %)	17 (77.3 %)	13 (65.0 %)
**Serum lipids (mmol/L)** ^a^				
Total blood cholesterol	4.54 ± 0.82	4.86 ± 0.88	4.76 ± 0.77	5.30 ± 1.47
Total LDL-C	2.66 ± 0.67	3.09 ± 0.79	2.76 ± 0.69	3.19 ± 1.13
Total HDL-C	1.21 ± 0.33	1.08 ± 0.22	1.01 ± 0.22	1.01 ± 0.25
Triglycerides	1.58 ± 0.36	1.63 ± 0.41	3.14 ± 0.70	3.44 ± 1.03

^a^Results are mean ± SD of (*n*) determinations

^b^Numbers in parentheses are  % of the total in each group

### Primary end point measure: correction of LC n-3 PUFA deficiency

**Fig. 2:**
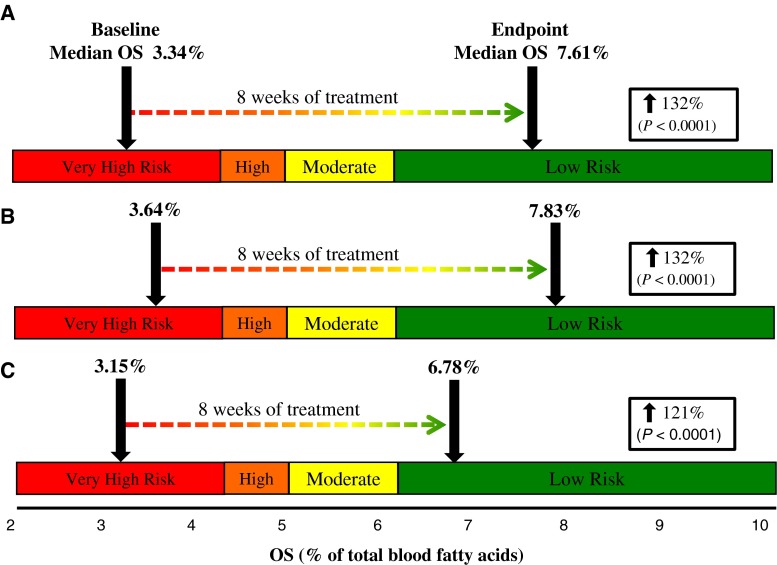
Change in blood median OS (primary endpoint) following 8 weeks of treatment with 6:1-OM3. **A** Total 6:1-OM3 treated (*n* = 56) in both Cohorts, **B** Cohort 1 (baseline TG 1.02–2.25 mmol/L, *n* = 36), and **C** Cohort 2 (baseline TG 2.26–5.65 mmol/L, *n* = 20). Percentage increases (*box*) represent a significant change (*P* < 0.0001) in placebo-adjusted median OS from baseline to week 8. Risk quartiles were adapted from previously published data [10]. For abbreviations, see text

The data presented in Fig. 2 show median OS levels of all 6:1-OM3 study subjects (*n* = 56) and of subjects in each Cohort group at baseline and after treatment for 8 weeks, when superimposed on to a previously defined risk quartiles Ref. [10]. Figure shows that regardless of baseline TG status, the median baseline OS was similar, and treatment with 6:1-OM3 effectively corrected LC n-3 PUFA deficiency by raising the median OS significantly in combined Cohorts (132 %, *P* < 0.0001) or in Cohort 1 & 2 (132 % & 121 %, *P* < 0.0001, respectively). The baseline median OS levels and the extent of correction in two Cohorts appeared to be dependent upon initial baseline TG levels (i.e., the higher the baseline TG, the lower the OS and lesser the correction with LC n-3 PUFA). The OS and OI indices increased in a similar fashion after 8 weeks of treatment (Table 2). As expected, both of these indices did not change in the placebo group, indicating that the dietary intake of the study participant were in compliance with the study protocol and did not change during the course of the trial.

**Table 2:** Summary of primary and secondary endpoint measures

	Placebo			6:1-OM3			Placebo-adjusted % change from baseline	
	Baseline	Week 8	% Change baseline-week 8	Baseline	Week 8	% Change from baseline	Placebo versus 6:1-OM3	*P*
**Cohort 1**								
**Primary end point**								
Omega-score (*n* = 32, 32, 36, 36)	3.38 (1.29)	3.60 (1.03)	0.3 (27.8)	3.64 (1.38)	7.83 (2.77)	132.2 (101.3)	131.9	<0.0001
Omega-3 index (*n* = 32, 32, 36, 36)	3.56 (1.26)	3.61 (1.11)	3.2 (28.5)	3.81 (1.31)	7.83 (2.58)	127.3 (82.5)	124.1	<0.0001
**Secondary end points**								
TG (mmol/L) (n = 32, 32, 36, 36)	1.48 (0.58)	1.59 (1.12)	2.0 (34.0)	1.75 (0.84)	1.54 (0.63)	−6.0 (27.3)	−8.0	0.11
Total Cholesterol (mmol/L) (*n* = 32, 32, 36, 36)	4.65 (1.03)	4.75 (1.51)	2.2 (11.5)	4.84 (1.24)	5.07 (1.12)	2.3 (17.8)	0.1	0.50
VLDL-C (mmol/L) (*n* = 32, 32, 36, 36)	0.69 (0.21)	0.65 (0.27)	0.0 (22.2)	0.66 (0.26)	0.67 (0.23)	−1.9 (28.3)	−1.9	0.34
IDL-C (mmol/L) (*n* = 32, 32, 36, 36)	0.39 (0.14)	0.36 (0.21)	−9.3 (31.8)	0.45 (0.22)	0.40 (0.24)	−6.4 (43.4)	3.0	0.54
LDL-C (mmol/L) (*n* = 32, 32, 36, 36)	2.67 (0.72)	2.67 (1.03)	1.4 (17.2)	2.99 (1.16)	3.32 (1.08)	7.0 (25.9)	5.6	0.12
HDL-C (mmol/L) (*n* = 32, 32, 36, 36)	1.18 (0.42)	1.15 (0.42)	0.0 (14.1)	1.06 (0.27)	1.09 (0.26)	−2.2 (13.5)	−2.2	0.42
NHDL-C (mmol/L) (*n* = 32, 32, 36, 36)	3.22 (0.96)	3.30 (1.19)	3.2 (14.9)	3.74 (1.23)	4.10 (1.04)	2.5 (21.5)	−0.8	0.22
Blood glucose (mmol/L) (*n* = 23, 23, 27, 27)	5.10 (1.30)	5.60 (1.70)	−3.9 (36.4)	5.30 (1.10)	5.40 (1.00)	4.3 (24.4)	8.1	0.89
AA:EPA (*n* = 32, 32, 36, 36)	21.00 (11.80)	24.00 (13.50)	7.0 (45.8)	21.20 (13.40)	3.00 (2.30)	−85.6 (20.1)	−93.0	<0.0001
n-6:n-3 (*n* = 32, 32, 36, 36)	9.70 (2.3)	9.80 (2.60)	3.6 (29.5)	9.60 (2.90)	4.60 (1.60)	−52.2 (17.9)	−48.6	<0.0001
hs-CRP (mg/L) (n = 32, 32, 36, 36)	3.40 (7.4)	1.90 (5.40)	−2.7 (58.4)	2.40 (4.20)	2.20 (4.40)	0.0 (73.3)	2.7	0.23
Apo-B (g/L) (*n* = 32, 32, 36, 36)	0.90 (0.18)	0.92 (0.29)	2.5 (13.2)	1.02 (0.25)	1.08 (0.31)	5.8 (17.7)	3.3	0.32
Apo-A (g/L) (*n* = 32, 32, 36, 36)	1.38 (0.27)	1.37 (0.33)	1.5 (8.1)	1.32 (0.18)	1.32 (0.17)	−2.0 (9.1)	−3.4	0.14
Apo-B:Apo-A (*n* = 32, 32, 36, 36)	0.70 (0.20)	0.60 (0.20)	−0.7 (18.3)	0.80 (0.20)	0.80 (0.30)	6.5 (19.1)	7.2	0.11
**Cohort 2**								
**Primary end point**								
Omega-score (n = 22, 22, 20, 20)	3.36 (1. 61)	3.43 (1.09)	−2.0 (32.2)	3.15 (0.90)	6.78 (4.30)	118.9 (114.4)	120.9	<0.0001
Omega-3 index (n = 22, 22, 20, 20)	3.54 (1.53)	3.61 (1.27)	−2.9 (26.5)	3.34 (0.89)	6.80 (4.60)	109.1 (97.7)	112.0	<0.0001
**Secondary end points**								
TG (mmol/L) (n = 22, 22, 20, 20)	2.98 (0.72)	2.99 (1.41)	0.4 (45.0)	3.10 (1.31)	2.03 (1.53)	−47.4 (27.6)	−47.8	0.0005
Total Cholesterol (mmol/L) (*n* = 21, 21, 20, 20)	4.82 (1.32)	4.63 (1.19)	2.9 (10.7)	4.79 (1.72)	5.14 (1.85)	4.4 (11.3)	1.5	0.36
VLDL-C (mmol/L) (*n* = 22, 22, 20, 20)	0.93 (0.27)	0.92 (0.18)	0.0 (17.4)	1.05 (0.44)	0.75 (0.32)	−30.2 (26.4)	−30.2	0.0023
IDL-C (mmol/L) (*n* = 21, 21, 20, 20)	0.47 (0.18)	0.47 (0.18)	0.0 (27.9)	0.49 (0.27)	0.51 (0.33)	0.0 (28.0)	0.0	0.87
LDL-C (mmol/L) (*n* = 22, 22, 20, 20)	2.64 (0.78)	2.69 (0.85)	1.7 (21.8)	2.97 (1.64)	3.38 (1.45)	13.0 (21.5)	11.3	0.12
HDL-C (mmol/L) (*n* = 22, 21, 20, 20)	1.01 (0.30)	0.96 (0.32)	−5.7 (12.1)	1.01 (0.37)	1.01 (0.31)	3.4 (24.7)	9.1	0.0069
NHDL-C (mmol/L) (*n* = 21, 21, 20, 20)	3.70 (1.10)	3.81 (1.05)	3.7 (13.1)	3.82 (1.84)	4.03 (2.02)	1.5 (13.5)	−2.2	0.68
Blood glucose (mmol/L) (*n* = 14, 14, 10, 11)	5.60 (3.00)	5.60 (2.40)	−4.7 (12.2)	5.40 (2.00)	5.60 (0.8)	4.3 (22.3)	8.9	0.23
AA:EPA (*n* = 22, 22, 20, 20)	15.00 (7.10)	14.10 (6.30)	11.3 (25.5)	16.00 (12.80)	3.50 (5.80)	−75.0 (23.0)	−87.0	<0.0001
n-6:n-3 (*n* = 22, 22, 20, 20)	9.30 (3.30)	9.04 (1.70)	2.3 (35.3)	9.10 (2.90)	5.10 (2.90)	−43.3 (27.8)	−45.5	<0.0001
hs-CRP (mg/L) (n = 22, 22, 20, 20)	3.40 (4.00)	3.30 (2.80)	4.8 (76.3)	1.60 (5.70)	1.90 (4.50)	3.6 (33.0)	−1.3	0.97
Apo-B (g/L) (*n* = 22, 20, 20, 20)	1.00 (0.22)	0.99 (0.25)	1.7 (13.9)	1.05 (0.40)	1.08 (0.45)	−0.4 (14.8)	−2.1	0.74
Apo-A (g/L) (*n* = 21, 20, 20, 20)	1.33 (0.18)	1.31 (0.19)	−1.4 (7.3)	1.35 (0.34)	1.38 (0.29)	−2.4 (12.3)	−1.0	0.58
Apo-B:Apo-A (*n* = 22, 20, 20, 20)	0.70 (0.20)	0.70 (0.20)	1.4 (11.3)	0.80 (0.20)	0.80 (0.30)	3.2 (15.6)	1.8	0.96

Data are presented as median (interquartile range)

**Fig. 3:**
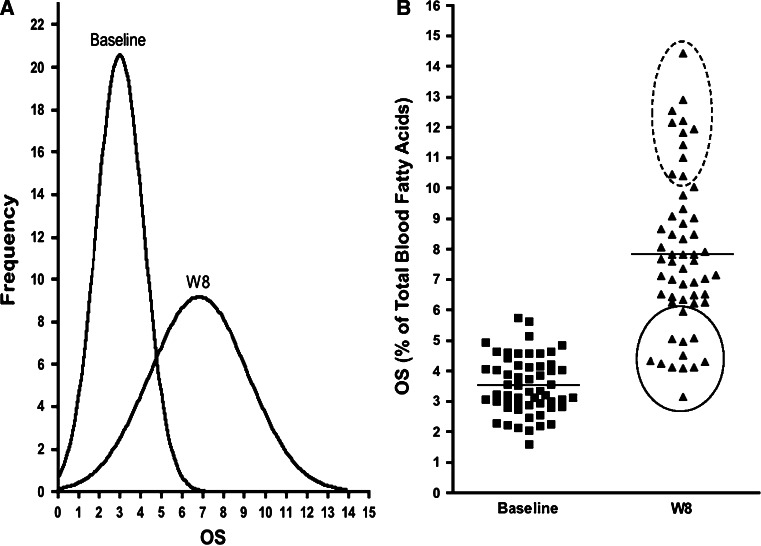
Change in OS (primary endpoint) at baseline and upon 8 weeks of treatment with 6:1-OM3 (*n* = 56). **A** Frequency distribution plot of panel B subjects with a broad distribution of OS due to weak versus strong responders. **B** Scatter plot of OS in 6:1-OM3 treated. Weak- and strong responders are *circled* with *solid* and *dotted lines*, respectively and horizontal lines at baseline and 8 week represent median values

The frequency distribution curve (Fig. 3a) shows that the correction of LC n-3 PUFA deficiency was subject dependent as a broader OS distribution was observed following 6:1-OM3 treatment than was observed at baseline. This is more evident in a scatter plot (Fig. 3b) where 12 out of 56 subjects (21 %) were found to be weak responders (solid line circle) as their mean OS changed by only 50 % (OS 3.0–4.5 %) as compared to 132 % for the combined group (*n* = 56). Another 11 out of 56 subjects (20 %) responded strongly (dotted line circle) to treatment where the greatest increase of 183 % (OS > 10.2) in OS was observed. The weak responders tend to be associated more with hypertriglyceridemic subjects (*n* = 8, Cohort 2), than with normal to marginally high TG subjects (*n* = 4, Cohort 1) while the strong responders belonged more to Cohort 1 (*n* = 8) than to Cohort 2 (*n* = 3). The average responders (*n* = 33), comprised 59 % of the treated subjects, showed a 121 % increase in OS with the majority (73 %) belonging to the normal to marginally high TG group.

### Secondary end point measures: effect of treatment on lipid profile

**Fig. 4:**
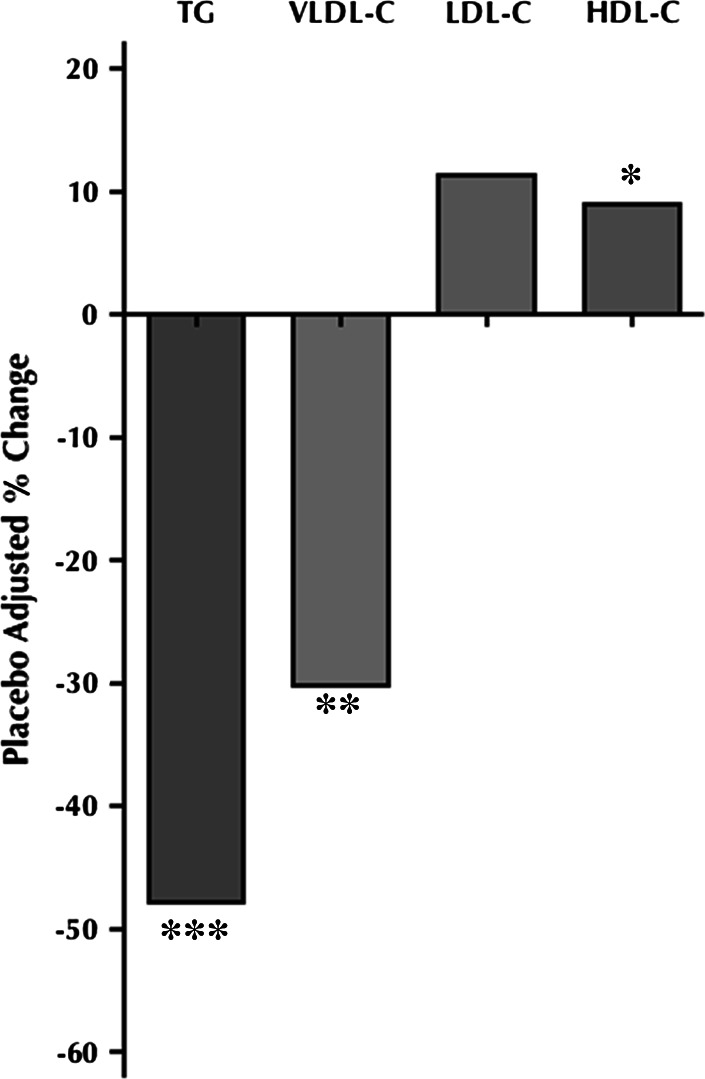
Percent change from baseline to week 8 of placebo-corrected blood lipid levels in Cohort 2 subjects (baseline TG 2.26–5.65 mmol/L). Analysis was on the per protocol population. ^*^9.05 % increase (*P* = 0.0069); ^**^30.20 % decrease *(P* = 0.0023); ^***^47.75 % decrease *(P* = 0.0005)

**Fig. 5:**
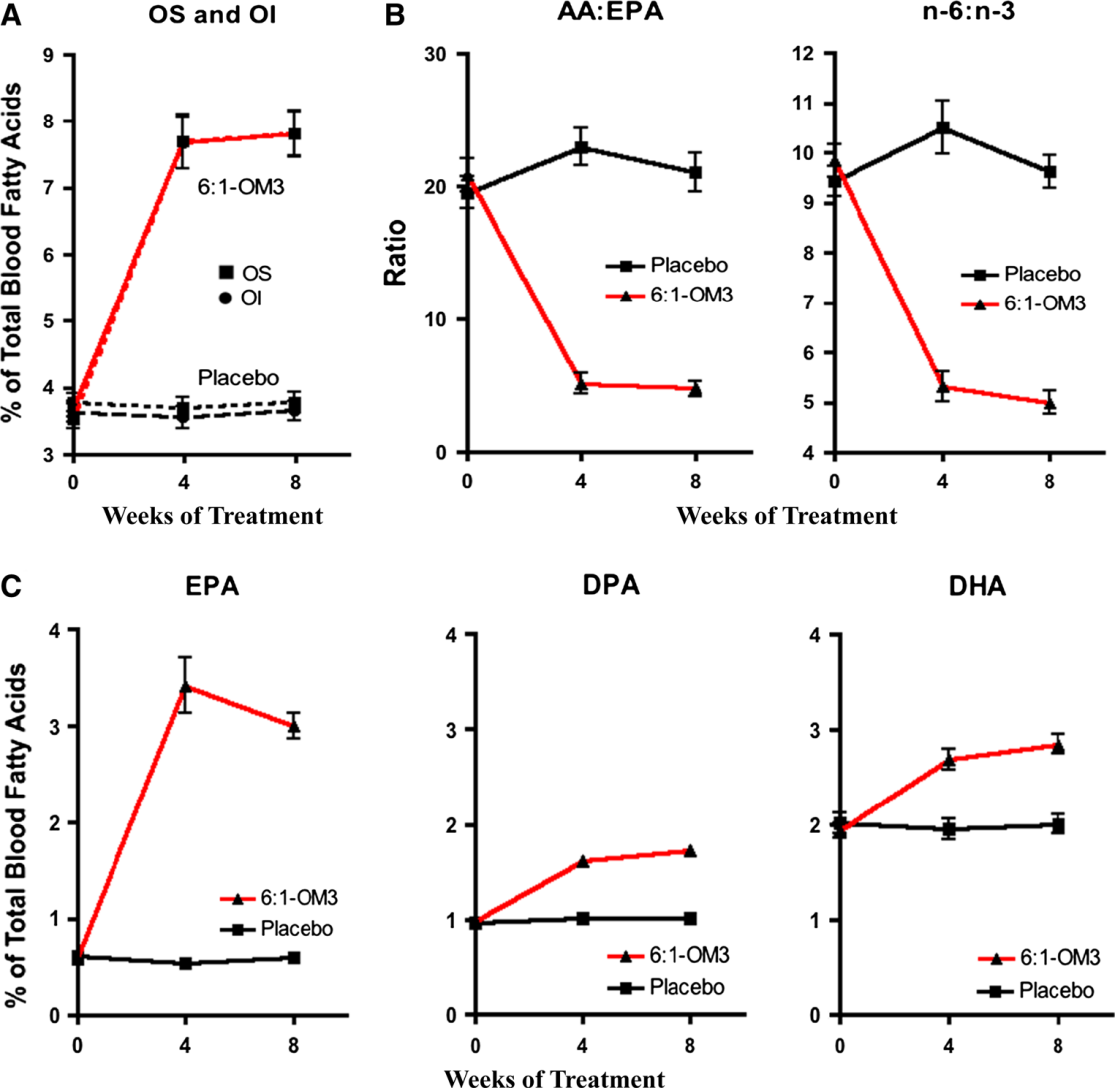
Time-course change in OS, OI, AA:EPA and n-6:n-3 ratios, and in individual LC n-3 PUFA components (*n* = 56) in both Cohorts upon 8 weeks of treatment with 6:1-OM3 and with placebo. **A** Changes in OS and OI. **B** Changes in inflammatory indicators, and **C** Changes in individual blood EPA, DPA, and DHA levels. All changes were significant (*P* < 0.0001) following 4 and 8 weeks of treatment. For abbreviations, see text

Table 2 and Fig. 4 illustrate the effects of 6:1-OM3 treatments on the secondary end points in both Cohorts. Cohort 2 subjects showed significant reduction in placebo-adjusted median levels of TG (47.8 %, *P* = 0.0005), and of VLDL-C (30.2 %, *P* = 0.0023), a significant increase in HDL-C levels (9.1 %, *P* = 0.0069), without any significant change in LDL-C (11.3 %, *P* = 0.1164) or in other secondary endpoints, including BMI, blood pressure, hs-CRP, apolipoproteins, and glucose. Arachidonic acid to EPA ratio and n-6:n-3 ratio significantly decreased (87.0 %, *P* < 0.0001; 45.5 %, *P* < 0.0001, respectively) in the 6:1-OM3 group as compared to placebo (Fig. 5b). Unlike Cohort 2, study subjects in Cohort 1 only showed a downward (−8.0 %), but a non-significant trend (*P* = 0.1140) in TG levels upon 6:1-OM3 treatment (Table 2). Similarly, lipoprotein-associated cholesterol in all fractions or other end points measures listed in table did not change significantly upon either treatment with the exception of AA:EPA and n-6:n-3 ratios. Both AA:EPA and n-6:n-3 ratios were reduced significantly by 93.0 % (*P* < 0.0001) and 48.6 % (*P* < 0.0001), respectively and this reduction was comparable to that of Cohort 2 as described above (Table 2, Fig. 5b).

The time course of changes in OS and OI were similar, reached a plateau after 4 weeks of treatment (Fig. 5A), and did not change significantly thereafter. The differential changes in individual LC n-3 PUFA are illustrated in Fig. 5C. All three PUFA, regardless of Cohort designation significantly changed after 4 weeks of treatment, reached a plateau and increased very little thereafter. The relative increases were very high for EPA (408 %), followed by DPA (75 %) and DHA (47 %) at the study endpoint. As expected, these components in the placebo group remained unchanged. The time course of changes in the AA:EPA and n-6:n-3 ratios were also similar (Fig. 5B), and both of these inflammatory indices decreased significantly (82 % and 51 % respectively,  *P* < 0.0001) upon 8 weeks of 6:1-OM3 treatment.

### Safety

No serious adverse events related to the study treatment were observed (Table 3). The data shown includes TEAE with an incidence of >3 % across treatment groups, irrespective of Cohorts. All TEAE observed were transient, mild in nature and did not require any treatment.

**Table 3:** Safety profile (TEAE in >3 % of study subjects)^*^

	Placebo	6:1-OM3	Total
	*n* = 54	*n* = 56	*n* = 110
Fishy burp	0 (0.0 %)	3 (5.4 %)	3 (2.7 %)
Flatulation	0 (0.0 %)	2 (3.6 %)	2 (1.8 %)
Nausea	3 (5.6 %)	2 (3.6 %)	5 (4.5 %)

^*^Number (and percentage) of patients experiencing TEAE in each group during the course of the study. TEAE: Treatment-emergent adverse events

## Discussion

In this study, we investigated the efficacy of a novel 6:1-OM3 formulation to correct LC n-3 PUFA nutritional deficiency and determined concomitant improvement in lipid profiles in subjects with one or more risk factors for CVD. Due to the highly variable demographic (geographical, cultural) dietary intakes of US population it is difficult to assign a “normal” blood level or assign cut-off value that indicate nutritional LC n-3 PUFA deficiency or insufficiency, as the words often used previously for other fatty acids [38, 39] expressed as blood OS or OI numbers. Harris et al. [13, 40] elegantly discussed the need for establishing “dietary reference intakes” for EPA and DHA and summarized the evidence for the proposed cut-off points for the OI based upon data from earlier studies. Earlier work by Albert et al. [10], showed an inverse relationship between low OS quartile and the increased incidence of sudden cardiac death. Mozaffarian et al. [12] recently authenticated the inverse relationship among low levels of individual or combined EPA, DHA, or DPA and the increased incidence of total mortality associated with CVD. Taken together, these studies shed some light on target OS or OI levels that offer the greatest protection, as described above. It was suggested that an OS of >6.1 % [10, 12] or OI of >8.0 % [8, 11] reduce CVD-related risk to the greatest extent, and these “target values” could be considered as optimal for CVD patient health. Based upon these studies, we chose to use an OS value of 6.1 % as a cut-off number to select study subjects designated as LC n-3 PUFA deficient. The data presented here show that 89 % of the 655 randomly selected screened subjects fell well below the 6.1 % OS cut-off value discussed above. The data confirm that the prevalence of LC n-3 PUFA deficiency in the US population is alarmingly high, putting subjects at greater risk for CVD complications and premature death, as was stipulated earlier [9]. Further, the median blood OS value in our screened subjects (*n* = 655) was 4.48 %, which is similar to the mean OS of 4.82 % that was determined earlier in participants who died suddenly from cardiac causes without prior evidence of CVD [10]. Thus the evaluation of LC n-3 PUFA status can be used as a valuable tool to select study subjects for RCT investigating the effects of LC n-3 PUFA supplementation, to determine if an adequate dose is provided to subjects to overcome LC n-3 PUFA deficiency, and to investigate its effect on the study outcome. A shift of focus from LC n-3 PUFA nutritional “intake” to the measurement of LC n-3 PUFA “levels”, at pre- and post-treatment should be a key consideration for studies evaluating the effectiveness of LC n-3 PUFA treatment.

In the present study, the median baseline OS of the subjects (*n* = 56) was 3.34 %, lying in the highest risk quartile (OS 2.12–4.32 %) for CVD death [10]. This LC n-3 PUFA deficiency was effectively corrected raising OS to 7.61 % (+132 %) within 8 weeks of treatment, achieving levels that lie in the lowest risk quartile (OS 6.08–10.02 %), corresponding to a risk reduction of about 81 % for sudden cardiac death [10]. The changes in both OS and OI observed in our study were similar and gave comparable information on the LC n-3 PUFA status as those reported previously [13]. However, our study was the first to report subject dependent differential effect of LC n-3 PUFA treatment. Here we identified weak responders where the mean increase in OS was considerably, and significantly less (50 %) than the mean increase of 132 % that was observed with the combined group (*n* = 56). Nevertheless, such an increase in OS still reduced the risk for sudden death by 45 % as described previously [10]. The majority of these subjects belonged to the hypertriglyceridemic group with an average BMI of 35.5 (obese). We also observed strong responders who showed significantly greater changes in OS with resulting endpoint OS levels of >10.2 %. These subjects belonged to the normal to marginally high TG group with a significantly lower average BMI of 27.5 (overweight), compared to the weak responders. The majority of the subjects (59 %) were average responders with a 121 % increase in OS levels and 73 % of these belonged to the Cohort 1. The numbers of subjects on medication were similar among Cohorts (50 % Cohort 1 and 45 % Cohort 2), although both weak and strong responders had different drug regiments. Further studies are needed to substantiate this novel finding and to investigate the mechanism associated with these differential responses, as the sample size of the current study was relatively small and the duration of the treatment was short. However, it appears that the individual personal health, particularly baseline TG levels, BMI, and the concomitant use of drugs may play a role in the differential absorption and metabolism of fish oils.

The Western Diet is abundant in n-6 fatty acids, and is relatively low in n-3 PUFA, resulting in an overall pro-inflammatory status mediated by AA versus EPA driven processes that have direct effects on the vascular endothelium, platelets, and immune cells [41, 42]. Although the baseline AA:EPA ratio in relation to CVD has been described previously, very few studies have shown pre- and post-treatment changes. Here we report a substantial decrease in the n-6:n-3 and AA:EPA ratios (51 % and 82 %, respectively), upon 8 weeks of treatment. These decreases are less but comparable (54 % and 99 %, respectively), to those observed recently with an EPA-only formulation in patients with higher TG levels (>5.65 mmol/L) and for a longer (12 weeks as compared to 8 weeks) treatment period [43]. Since inflammation plays a significant role in the pathogenesis of CVD [44], and the presence of a low AA:EPA ratio has been described to prevent the onset or progression of CVD [45], significant decreases in both AA:EPA and n-6:n-3 in the present study are indicative of an improvement in the inflammatory status of the subjects. Further studies elucidating the downstream consequences of these low ratios are currently being undertaken.

With respect to secondary endpoint measures, the data presented here differ considerably from those reported in other RCTs that utilized LC n-3 PUFA formulations with similar purity (>90 %) and dose (4 g/day), but different EPA:DHA ratios (1.2:1 & EPA-only), study duration, and patient selection. In the present study, subjects with baseline TG levels between 2.26 and 5.65 mmol/L, had a significant decrease in TG (48 %), and VLDL-C (30 %), after 8 weeks of treatment. Placebo-corrected post-treatment HDL-C levels improved a little and LDL-C levels did not significantly change. In a comparable study of patients with high TG levels (2.26–5.65 mmol/L), treatment with an EPA-only formulation resulted in a less efficacious reduction in TG (21 %), and VLDL-C (24 %), over a longer treatment period [46] than the present study. Treatment of patients with >500 mg/dL baseline TG level with the same EPA-only formulation, resulted in a greater decrease in TG (33 %) and VLDL (29 %) than that was observed previously [26]; however, the reduction was still less pronounced than reported here and over a shorter period of treatment. No significant changes in HDL-C and LDL-C levels were observed in those studies, while in our study, only placebo-corrected improvement (9 %) in HDL-C was observed. When an EPA:DHA formulation of 1.2:1 was utilized in another study of patients with >5.65 mmol/L TG, a greater TG (52 %) and VLDL-C (41 %) reduction was observed after 16 weeks of treatment, but with a significant increase of up to 49 % in LDL-C [47, 48]. The differences in the secondary endpoint parameters in the above studies could be attributed to the differential properties of EPA and DHA [49], and the utilization of different LC n-3 PUFA formulations with variable ratios. Both EPA and DHA are known to reduce TG levels. However, DHA alone can contribute to an increase in LDL-C levels, and EPA alone is less efficacious at lowering TG and VLDL levels as was observed in the above studies that investigated patients with an EPA:DHA ratio of 1.2:1, or EPA-alone formulations. The differences in the present study could be attributed to the use of an optimal EPA:DHA ratio. This 6:1-OM3 formulation has been shown to produce maximum and sustained vasodilatory effects in isolated perfused porcine coronary artery rings than some other known LC n-3 PUFA preparations [31]. Taken together, on equivalent dose, purity, and patient selection (equivalent baseline TG levels), a 6:1-OM3 formulation shows the greatest TG, and VLDL-C reduction, without significant increase in LDL-C levels, and in a shorter treatment period.

The effects of LC n-3 PUFA supplementation on normal to marginally high TG subjects (1.02–2.25 mmol/L) considerably differ from their effects on subjects with high TG (2.26–5.65 mmol/L), or very high TG (> 5.65 mmol/L). To our knowledge, little is known about the impact of LC n-3 PUFA in subjects with normal to marginally high TG. Although comparable RCT data in such group is not available, the present study shows a downward, but non-significant effect on TG and VLDL-C levels upon treatment, without affecting other lipids. However, the correction of LC n-3 PUFA deficiency, as measured by OS showed a similar increase of 132 %, with significant and equal reduction in the n-6:n-3 and AA:EPA ratios (−48.6 % and −93 %, respectively) when compared with hypertriglyceridemic subjects. This suggests that beneficial effects of LC n-3 PUFA are more than simply reduction in serum TG levels. Other secondary endpoints such as apo-A, apo-B, hs-CRP, BMI, and blood pressure, did not reach statistical significance, irrespective of the Cohorts and may require longer treatment regiment to claim differences.

From the above discussion, it is apparent that the efficacy of LC n-3 PUFA treatment of hypertriglyceridemia appears to be dependent upon the severity of dyslipidemia; the higher the baseline TG level, the greater the potential percent reduction upon treatment. Also, the efficacy of a formulation to improve lipid profiles, inflammatory indices, and vasodilatory effects appear to be EPA:DHA ratio dependent. To this end, higher circulating DHA levels have been shown to be associated with lower incidence of atrial fibrillation and play an important role in the protection from sudden death from cardiac causes [12, 50]. Whether related or not, protection from sudden death was observed in the landmark GISSI study, that utilized a formulation containing DHA in contrast to the JELIS study that utilized an EPA-alone formulation, where this protection was not observed, [18, 19]. The greatest protection from cardiovascular mortality including coronary heart disease, arrhythmic, and non-arrhythmic mortality has been described to be associated with the highest plasma phospholipid DHA levels, while non-fatal myocardial infarction mortality was associated with the highest plasma phospholipid EPA levels [12]. Thus, it appears that inclusion of low amounts of DHA with EPA in LC n-3 PUFA formulations offers the greatest potential for correction of LC n-3 PUFA deficiency-associated CVD risks, without increasing LDL-C. In addition, increased levels of DPA have been associated with lower risk (47 %) for stroke death and may, together with EPA, contribute to plaque stability [51].

The data presented here show that a 6:1 EPA:DHA ratio appears to be optimal for correcting LC n-3 PUFA nutritional deficiency, with concomitant positive effects on lipid profiles and on inflammatory indices. As mentioned by others [27, 28], the present study also highlights the need to consider LC n-3 PUFA dose and EPA:DHA ratio in the treatment formulation, patient selection, as well as the importance of pre- and post-treatment measurement of OS and/or OI. Further studies with lager patient enrollment and of longer treatment duration are needed to investigate beneficial, and differential effects of LC n-3 PUFA intake on heath, inflammation, and mortality due to CVD.

## Abbreviations

LC n-3 PUFA: Long chain omega-3 polyunsaturated fatty acids
n-3 & n-6 fatty acids: Omega-3 & omega 6 fatty acids
AA: Arachidonic acid
EPA: Eicosapentaenoic fatty acid
DHA: Docosahexaenoic acid
FAME: Fatty acid methyl-esters
6:1-OM3: LC n-3 PUFA formulation of >90 % purity with a 6:1 EPA and DHA ratio
OI: Omega-3 Index, combined red blood cell levels of EPA and DHA as a % of total fatty acids
OS: Omega-Score™, combined blood levels of EPA, DHA and DPA as a % of total fatty acids
MAP: Mean arterial pressure
BMI: Body mass index
CVD: Cardiovascular disease
CRO: Contract research organization
hs-CRP: High sensitivity C-reactive protein
RTC: Randomized controlled trial
TEAE: Treatment-emergent adverse events
